# Editorial for Special Issue: Advances in Sedentary Behavior Research and Translation

**DOI:** 10.3934/publichealth.2017.1.33

**Published:** 2017-01-18

**Authors:** Stuart J.H. Biddle, Jason Bennie

**Affiliations:** Institute of Sport, Exercise & Active Living (ISEAL), Victoria University, Footscray Park, Melbourne, VIC 8001, Australia

Sedentary behaviour—essentially low energy sitting time in waking hours—has emerged as an important topic in public health over the past decade or so. Although Morris and colleagues [1] analysed health outcomes of active versus seated occupations over 60 years ago, it was not until studies of TV viewing in children in the 1980s [2] that researchers started to recognise “too much sitting” as a potentially important health behaviour. Even then the rapid rise in the study of sedentary behaviour was not so evident until the early 2000s [3]–[5]. Studies on screen viewing (TV and computers), sitting at work and school, and sitting in cars have all emerged over this period, as well as a general recognition that high levels of sitting may have detrimental effects on health, and possibly be independent of levels of moderate-to-vigorous physical activity (MVPA). In the past 10–15 years there has been an exponential increase in papers addressing sedentary behaviour from the perspective of sitting, noting that many exercise physiologists still use the word “sedentary” incorrectly by referring to those not meeting a criterion level of “sufficient” physical activity.

In this special issue of the journal we encouraged papers that could demonstrate innovation and thus advance the field. We also wanted to see more papers on translation of evidence. The behavioural epidemiology framework has five main phases: (i) the study of health outcomes; (ii) measurement of the behaviour; (iii) correlates of behaviour; (iv) interventions to change behaviour; and (v) translation of findings [6]. There was initial emphasis in the field of sedentary behaviour on measurement [7], particularly regarding the prevalence of sedentary behaviour, the correlates of sedentary behaviour [8], as well as health outcomes, such as adiposity [9]. This was followed by a greater focus on behaviour change as interventions emerged in various settings, including the home, work, and schools [10],[11]. In comparison, little has been said about translation of findings.

**Figure 1.**
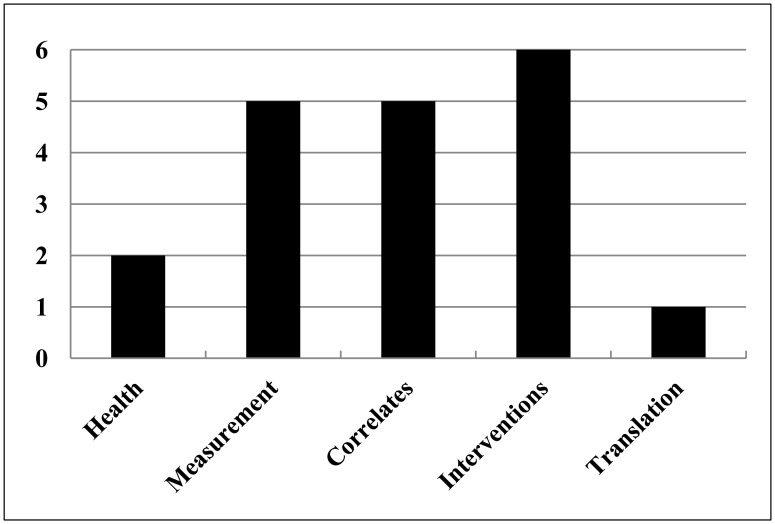
Distribution of key topics across the phases of the behavioural epidemiological framework.

In this special issue, we published 19 papers led by authors from five countries (Australia, Canada, Finland, UK, and USA). An approximate allocation of the main topic of each paper to one of the five phases of the behavioural epidemiology framework shows that papers were more likely to address measurement, correlates and interventions (see Figure 1). It was disappointing not to attract more overtly translational papers, although some did address translational issues alongside their intervention findings.

Regarding innovation and advancing the field, papers addressed a number of novel topics and approaches, including health outcomes through muscle inactivity time assessed via electromyography (EMG) [12], measurement through mixed methods lifelogging technology to capture behavioural context [13], analysis of large data sets [14], assessing children and their parents [15], assessment of context and health outcomes [14], correlates of weather variation and urban design [16], psychological constructs of motivation and attitudes [17],[18], interventions in schools [19],[20], scaling up of an intervention for translation [21], as well as qualitative studies on people's views concerning sedentary behaviour change [22],[23]. There was greater interest in the study of adults, including older adults, than youth. This may reflect the much greater interest in youth in prior literature and the need for a more balanced approach across the lifespan.

The measurement of sedentary behaviour remains a challenging field. The papers in this special issue used a multitude of approaches, including different wearable devices, self-report measures, experience sampling [18], qualitative approaches and cameras [13]. This field will continue to diversify with the advancements in technology, but self-reported behaviours and perceptions will remain an important area as we try to assess context and preferences for behaviour change. There are also many “subjective” decisions made in the implementation and analysis of wearable technology devices, hence it may be better to refer to these as wearable devices rather than “objective” measures.

Interventions, whether in the workplace or schools, were still mainly focussed on the provision of sit-to-stand desks. Such an environmental change can be successful in reducing sitting time, but more is needed on the acceptability and feasibility (e.g. cost) of such an intervention and whether simple alternatives are also possible. Some people will not work at a desk but still have high levels of sitting. Moreover, health outcomes need investigating where standing is compared more with different levels of movement [24]. Marshall and Merchant's [25] concept of different “behavioural typographies” is worthy of further exploration as some interventions will require the same task to be done in a different posture (e.g. standing), while some will need to substitute sitting with a more active behaviour that may mean the seated task is no longer possible. The paper in this special issue by Wennman et al. [14], for example, showed that not all types (measures) of sedentary behaviour were equally associated with cardiovascular risk. This has implications for interventions and what types of sedentary behaviours to target. For example, with the emergence of studies showing that those with high levels of MVPA are largely unaffected by high levels of sitting [26], do we focus only on MVPA or continue our efforts to promote physical activity alongside less sitting?

This special issue goes a small way to advancing the field, but there is much still to do across all phases of the behavioural epidemiology framework, but especially in documenting health outcomes of different sedentary and alternative behaviours, types and settings of interventions, and translation into practice. As Co-Editors of this issue we would like to thank the authors for submitting their papers and the reviewers for their efforts in undertaking peer review.
